# Threats and Opportunities When Using Chickens as a Model for Host–Microbiota Studies

**DOI:** 10.3390/microorganisms14061330

**Published:** 2026-06-13

**Authors:** Ivan Rychlik

**Affiliations:** Veterinary Research Institute, Hudcova 70, 62100 Brno, Czech Republic; rychlik@vri.cz; Tel.: +420-533331201

**Keywords:** chicken, hen, gut microbiota, age, hatchery

## Abstract

Millions of chicks are hatched daily in commercial hatcheries and due to ease of access and the large availability of chicks produced daily, such chicks have been accepted as a reference and control. Unfortunately, this is not a correct assumption. Chickens evolved to be hatched in nests and to remain in close contact with adult hens, which is important for the transfer of chicken-adapted microbiota from hens to offspring. In the absence of adult hens, chicks from hatcheries are colonised by microbiota of environmental origin. Forgetting this fact has led to many confounding conclusions, including a dogma on the age-dependent development of gut microbiota. In this sense, chicks from hatcheries represent a threat. However, if correctly perceived, the same chicks represent a unique opportunity for host–microbiota studies since there is no alternative animal model in which offspring free of any paternal influence are that readily available.

## 1. Introduction

Warm-blooded vertebrates are born or hatched essentially sterile but soon after birth they become colonised by complex microbiota, resulting in the formation of a metaorganism consisting of a similar number of host and microbial cells [1]. The colonisation starts as early as during birth and then develops quickly before a delicate balance between host and microbiota is established. However, before the balance is achieved, different disorders may occur. Since microbial densities are the highest in the gastrointestinal tract, dysfunctions of the gastrointestinal tract in newborn individuals are quite frequent.

When thinking of eukaryotic hosts as metaorganisms, it is also possible to consider gene counts. The genomes of humans, pigs, cattle or chickens consist of 20,000–25,000 genes [2,3,4,5]. In comparison, the intestinal tract of each of these hosts is colonised by approximately 1000 different bacterial species and, since the genome of an average gut anaerobe is around 3 Mbp [6] and an average prokaryotic gene size is around 1000 bp, each bacterium from the intestinal tract encodes around 3000 genes. The total gut microbiome consisting of genomes of 1000 different bacterial species therefore comprises approximately 3 × 10^6^ genes. When comparing this to the 2 × 10^4^ genes of the chicken, the gene number from the chicken gut microbiome dominates over that of the chicken by two logs and considerably contributes to the metabolic potential of its host. Moreover, the above mentioned numbers still underestimate the contribution of microbiota on host performance since (i) multiple strains of the same bacterial species with slightly different genomes may colonise the same host; (ii) total gut microbiota is formed not only by bacteria but also by viruses, phages, protozoa and fungi [7,8,9,10,11,12,13,14]; and (iii) microbial colonisation is found not only in the intestinal tract but virtually at any surface exposed to the external environment [15,16,17,18]. There is therefore no doubt that the composition of microbiota considerably affects host behaviour [19], body weight [20], circadian regulation and brain expression [21], movement patterns [22], or development of gut immune system [23].

## 2. Chickens as a Specific Model for Host–Microbiota Studies

Hatched chicks are not fed by parents and can forage on their own from the first moments of their life. Because of this, hatching can be accomplished without brooding eggs by hens. However, chickens evolved to be hatched in nests and to remain in contact with the hen and other members of the flock. Such contact is important for the colonisation of chicks by chicken-adapted microbiota where chicken-adapted microbiota means microbiota which is present in intestinal tract of adult chickens and can be easily transferred also to offspring. Ignoring this fact and relying on eggs incubated in hatcheries and the use of chicks from hatcheries for studying their microbial colonisation may lead to observationally correct but biologically mistaken conclusions; there many papers reporting on the age-dependent development of gut microbiota in chickens, needs for anaerobisation of intestinal tract environment by facultative anaerobes, or definition of mucosal microbiota analysing samples in one- or two-week-old chickens. In this sense, chicks from hatcheries represent a threat. However, if correctly perceived, the same chicks represent a unique opportunity for host–microbiota studies, as presented below.

Millions of chicks are hatched daily worldwide. Due to large-scale production, the cost of a single broiler chick is around 50 cents, which is incomparable with the cost of other animal models like Balb/C mice, rats, rabbits or even pigs. Daily broiler production also means daily availability of newly hatched chicks. However, there are two critical points which must be considered when performing experiments focused on chicken gut microbiota. The first one is the specific mode of chicken digestion and faecal excretion, and the second one is the fact that the vast majority of chicks are hatched in hatcheries in the absence of their parents. These points are critically discussed below, with somewhat unexpected conclusions sometimes being proposed in a slightly provocative style. Although the proposed conclusions are meant seriously, the ultimate purpose of this review is to force readers to think about the topic and modify their future experiments in a way that will allow them to check the hypotheses proposed in this paper.

## 3. Specifics of Chicken Digestion

**Figure 1:**
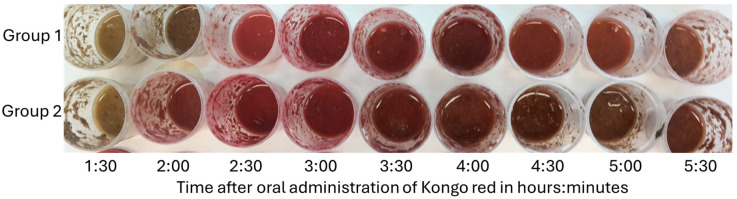
Transit time of digesta through chicken intestinal tract. Two different groups of 31-day-old egg-laying ISA Brown chickens (each consisting of 10 birds) were orally inoculated with 0.1 mL of Kongo red solution. Faecal droppings were collected from the floor in 30 min intervals and resuspended in water. Excretion of Kongo red was recorded 2–2.5 h after administration indicating the transit time of digesta through the chicken intestinal tract. Unpublished data from reference [24].

Chickens exhibit a specific mode of digestion. The majority of feed digested in the small intestine passes directly to the colon and is excreted to environment. In this case, the transit time from feed consumption to faecal excretion can be as short as 2 h (Figure 1).

**Figure 2:**
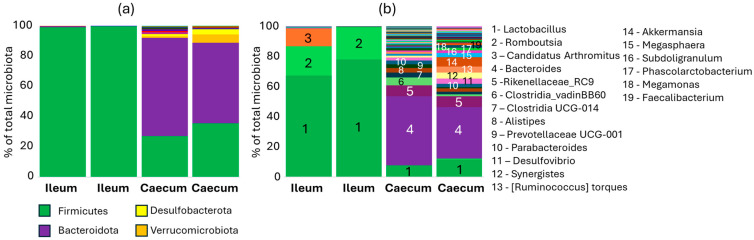
Microbiota composition in the ileum and caecum of adult hens. Two egg-laying hens, 40 weeks of age, were analysed for microbiota composition in the ileum and caecum by 16S rRNA sequencing. Since faecal droppings can be formed by either of these samples, collection of faecal samples without differentiation between ileal and caecal dropping leads to mixing up two different types of samples. Panel (**a**)—ileal and caecal microbiota composition at phylum level; panel (**b**)—ileal and caecal microbiota composition at genus level. This figure has been modified from reference [26].

Unlike humans or pigs, the chicken colon is short and contains minimal digesta. Due to the absence of extensive anaerobic fermentation in the colon, most of faecal droppings are of ileal origin and the microbiota composition from this type of faecal material corresponds to ileal microbiota. Small intestine colonisers such as *Lactobacillus*, *Romboutsia* or *Turicibacter* are therefore recorded in this type of faecal droppings [25,26,27,28]. Usually twice a day, ileal digesta is diverted to the caecum, which is then closed and anaerobic digestion and fermentation, similar to that in human or pig colon, proceeds for approximately 12 h [29,30,31]. The caecum is then opened, caecal digesta is ejected to the colon and excreted. The caecum is then filled with a new batch of ileal digesta and the whole process is repeated. Representatives from class Clostridia and phylum Bacteroidetes dominate in the caecum in adult chickens [32,33,34,35,36] and the microbial composition of caecal droppings is therefore completely different from that in ileal droppings (Figure 2).

Collecting faecal samples and calculating the average values from the samples which have different microbiota compositions leads to extremely confounding results [37]. Caecal droppings are ejected usually in the morning after overnight fermentation and at the end of the day, after caecal fermentation throughout the whole day. Ileal and caecal droppings can be visually differentiated, the former being of a lighter colour and more coarse structure. Caecal droppings are darker, of brownish to black colour and of homogenous structure due to fermentation. Despite this, if it is possible to sacrifice chickens and collect caecal and ileal digesta, this is recommended over faecal dropping collection. Although it can be argued that use of animals should be reduced, analysis of correct samples may save numbers of used animals due to providing relevant results at the very end.

## 4. Chicks from Hatcheries

**Figure 3:**
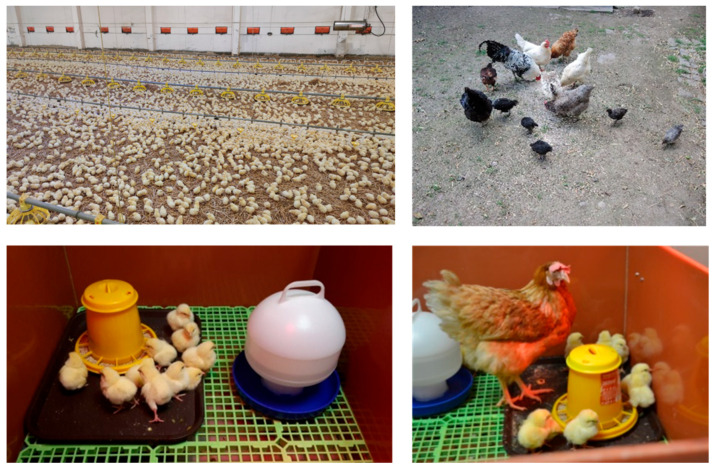
*Gallus gallus* evolved to be hatched in nests and not in hatcheries. Chickens represent an excellent model for host–microbiota studies. One only has to keep in mind that chickens from hatcheries, without any contact with adult birds, do not represent a control group. In fact, chicks from hatcheries deprived of any contact with parents represent an extremely experimental group.

The next characteristics specific to chickens is that essentially all studies use chicks that originate from hatcheries. Due to ease of access and the large availability of chicks produced daily, such chicks have been accepted as a reference and control. Unfortunately, this is a mistaken assumption. Chicks kept in contact with adult birds or inoculated with faecal or caecal extracts from adult birds should be considered as controls to model hatching in nests and natural transfer of chicken-adapted microbiota (Figure 3). This does not argue against the use of chicks from hatcheries as we need to know how gut microbiota of chicks develop in commercial settings. However, one must be aware that during the first days and weeks of life, the microbiota of these chicks reflects microbiota present in their environment and does not represent chicken-adapted microbiota. In addition, there are already enough studies of this type, and it is not necessary to produce additional ones since the principles will be the same; facultative anaerobes, like *E. coli* or enterococci, or aerotolerant lactobacilli initially colonise the chicken intestine [38,39]. Facultatively anaerobic metabolism is their advantage, but not because of their ability to cope with the residual oxygen in the intestinal tract of newly hatched chicks. Instead, the key is that they multiply and dominate in the external aerobic environment from which they infect naive chicks as the first ones. The anaerobic part of their metabolism then allows them to continue in proliferation also in the intestinal tract. With a minor delay, usually of 4 to 7 days, the first representatives of Clostridiales from families Lachnospiraceae, Oscillospiraceae and Ruminococcaceae appear [40,41,42] because these are present in external environment in the form of spores. Lastly, strictly anaerobic representatives of phylum Bacteroidetes appear [38,43,44]. A dogma on age-dependent colonisation of the chicken intestinal tract arose from such studies. Unfortunately, this conclusion is biologically mistaken and is a mere consequence of raising chickens in the absence of their parents [38,45,46,47]. The observed development has nothing in common with age and is a mere consequence of the probability with which chicks can be infected by particular bacterial taxa from the environment. This is the reason why facultative anaerobes replicating and dominating in the external environment (see Figure 4 in reference [15] for *E. coli* distribution across different sample types), and automatically continuing in their anaerobic metabolism, appear as the first ones. Aerotolerant bacteria being less abundant in the external aerobic environment and requiring additional time to refresh their metabolism appear second [48]. Spores of spore-forming bacteria are less abundant in external aerobic environment than facultative anaerobes or aerotolerant bacteria and need additional time for spore germination in the gut—which explains why these usually appear third [49,50,51,52]. The last colonisers of gut of chicks from hatcheries comprise strict anaerobes without any form of resistance to oxygen [53,54,55,56]. These are lowly abundant in the aerobic environment and have the lowest likelihood of newly hatched chicks coming in to contact with them. The proposed hypothesis on environment-driven colonisation need not be taken as an established fact but rather as a well-supported hypothesis. However, reports on the oral administration of faecal extracts or experiments with contact hens clearly show that this mode of development of chicken gut flora is not a must. There are multiple reports showing that chicks can be colonised by strict anaerobes and adult-type microbiota from the very first days of life [16,33,57,58,59,60] and such treatment results in chicken resistance to intestinal tract infections [61,62,63,64,65,66,67]. The resistance is induced within 24 h after treatment, showing that the interaction of gut microbiota with the chicken host is immediate [62,63]. Colonisation of chickens with individual strains is not effective [68] and, instead, complex microbial consortia are required [69]. Principles of early colonisations and pathogen exclusion are not fully understood. Complex microbiota affect the development of the gut immune system [23]; some gut microbiota members express antimicrobial substances like antimicrobial peptides [70] or type VI secretion system [71,72]. Despite this the most likely explanation is a mere numerical dominance of commensals competing for nutrients with opportunistic pathogens. These facts have been known for decades, though unlike the currently used term ‘faecal microbiota transplantation’, this phenomenon has been called ‘competitive exclusion’ in chickens [62,73,74,75,76].

The proposed hypothesis that the early colonisation of chickens depends on environmental availability and microbial characteristics, rather than age itself, is difficult to confirm experimentally. However, once all the above-mentioned facts are integrated, this becomes a quite likely explanation. This does not exclude the possibility that microbiota composition can be shaped during the chicken’s life, e.g., when the diet changes during different periods of chicken production or at the onset of egg lay, as a consequence of hormonal changes. Nevertheless, the initial colonisation of day-old chicks is indeed not associated with age, as many faecal transplantation experiments have already shown [77,78,79,80,81,82].

Experiments with chick colonisation by complex microbiota also show that it is not possible to define when the development of gut microbiota in chickens in commercial production is accomplished. If complex microbiota, e.g., contact donor hen, was provided on day 1, 10 or 30 to the flock, the development of gut microbiota would be accomplished a few days after day 1, 10 or 30, respectively. All of this is even more complicated in real life since microbiota is not introduced to the flocks by adult hens but by many different vehicles including human personnel, rodents, wild birds, insects, feed, drinking water or air dust. These vehicles will never transfer complete chicken-adapted microbiota but rather a few bacteria which are common to the vehicle and chickens. Access of the above-mentioned vehicles to farms may vary among farms and may differ in the same farm in different parts of year. A question on when the development of gut microbiota in commercially raised chickens is accomplished is therefore misleading and does not make any sense, and no correct answer to this question can be provided. Only general statements based on probability like the higher the biosecurity and zoohygienic standards, the longer time necessary for the development of gut microbiota in chickens, can be produced. Or in other words, experiments in academic settings with air conditioning and filtration, sterile feed and water and positive pressure animal houses may provide even more distorted results than observations in common poultry farms. But even in common poultry farms, the life span of broilers is usually too short to allow for the development of adult type gut microbiota.

## 5. How to Establish Biologically Relevant Control Chickens

There are multiple ways to set up a biologically relevant control group of chicks. Chicks from hatcheries can be exposed to fresh faecal material spread over the floor [83,84]. Newly hatched chicks can be orally inoculated by extracts from fresh faeces or ileal or caecal digesta [57,59,63]. Chicks can be kept in contact with adult birds [16,33] (Figure 3). The same can be achieved by administration of commercially available competitive exclusion products [75,85,86,87,88] or, quite importantly, by administration of individual pure cultures of gut anaerobes or their defined mixtures [68,69,89]. Since millions of chicks are hatched daily, the same number is slaughtered daily, which means that virtually unlimited numbers of chicken caeca or ilea with their contents are discarded in slaughterhouses daily and these can be used as sources of microbiota in controlled experiments, i.e., not in commercial settings because of potential biosafety risks. Although gut samples from egg layers or reproductive flocks are preferred over those from broilers, and this type of chicken is slaughtered less frequently than broilers, even in this case there is enough material available.

## 6. What Can Be Learnt if Chickens Are Correctly Used?

Faecal transplantation in chickens showed that not all microbiota members colonise the intestinal tract with the same efficacy. Following administration of caecal digesta from adult birds, different species of Bacteroidetes, Selenomonadales or strictly anaerobic Proteobacteria can be recorded in the caecum of treated chicks [33,57,69,89]. This has been confirmed by the administration of pure cultures when these taxa efficiently colonised, while representative of Firmicutes, including class Clostridia or genus *Lactobacillus*, belonged among the poor colonisers [68,90]. The same has been observed also in newborn piglets in which *Bacteroides* spp. but not lactobacilli efficiently colonised [91]. Quite likely, this is affected by the ability of bacteria from the intestinal tract to survive in the external aerobic environment—the better the survival outside the host, the lower the potential for persistent colonisation, and vice versa [92,93]. This also highlights the necessity to confirm whether the strains used as probiotics can persist in the treated chickens. Many studies report on lactobacilli-positive effects though do not test for the presence of the used strains in the intestinal tract. Our experience is that the most proficient Firmicutes persist in the intestinal tract for a maximum of 2–3 days and form up to 1% of total microbiota, which is in sharp contrast to *Bacteroides*, which can persist for more than 40 days and form more than 10% of total microbiota after a single dose oral administration on day 1 of life [69,94]. Colonisation of different *Bacteroides* species is so efficient that species otherwise characteristic for humans (*Bacteroides uniformis*, *B. vulgatus* or *B. dorei*) [95,96,97,98,99,100] can be found temporarily in the gut microbiota of chickens from hatcheries during the first weeks of life [68], likely as a consequence of human handling.

## 7. Passage Experiments and Generation Selection as Another Advantage of Chickens

Continuous availability of chicks from hatcheries allows for another type of experiment, which is difficult to set up in other vertebrates. Chickens can be used for the selection of microbial populations with desired properties via multiple generations. If the aim is to select microbiota supporting body weight increase, the first generation of chicks can be inoculated with the caecal extract of a donor hen and then at defined intervals—e.g., 1 week—caecal microbiota of the heaviest chick can be transferred to a new generation of newly hatched chicks. Selection criteria can be adopted according to the project aims, such as identification of microbiota members degrading less frequent feed components etc. Such selections, due to non-availability of naive animals free of any contact with parents, cannot be easily performed in other animals. One only has to be aware of the potential bias toward Bacteroidetes and Selenomonadales, which efficiently colonise chicken caecum, as they may overgrow and mask the performance of other microbiota members [69]. Why Bacteroidetes and Selenomonadales and several other taxa are such efficient colonisers is not known but we have observed that this behaviour is inversely dependent on survival in external aerobic environment—facultative anaerobes, aerotolerant and spore-forming bacteria usually poorly colonise the intestinal tract [69]. On the other hand, gut anaerobes without any form of resistance to the aerobic environment compensate such handicap with efficient colonisation [92,93].

## 8. Mucosal Microbiota

Due to specific digestion in the chicken caecum, mucosal microbiota in the chicken caecum is highly developed and differs from luminal microbiota. However, samples from adult hens rather than from one-week-old broilers must be analysed. If using samples from chickens of broiler age, mostly false positive microbiota members are identified as mucosal ones. If using caeca from adult hens, taxa usually reported as minority members in the chicken gut microbiota, e.g., *Mucispirillum* from phylum Deferribacteres, *Brachyspira* and *Treponema* sp. from Spirochaeta, together with *Helicobacter pullorum* and particular *Desulfovibrio* spp., dominate in caecal mucosa [72]. Such experience can be extrapolated to other vertebrate hosts and chicken caecal samples from animals slaughtered at commercial slaughter houses can be used for the development of protocols for the separation of luminal and mucosal microbiota.

## 9. Microbiota from the Chicken Environment

Large-scale production of chickens also allows for easy access to samples from their environment. There is a gap in knowledge on microbiota in hatcheries despite the fact that such bacterial species represent the first antigens to which just hatching chicks are exposed. Studies on litter microbiota or air dust microbiota are more frequent [101,102,103,104,105,106,107,108,109] but are commonly separated from comparison with skin, respiratory or intestinal tract microbiota of chickens. Some taxa characteristic for the litter, e.g., *Brachybacterium* or *Brevibacterium*, can be found on the skin or in the crop and are then mistakenly considered as characteristic for these organs [15,105]. Easy access to samples from the chicken environment thus may help with correct positioning of individual bacterial species to a particular type of samples.

## 10. In Vivo Gene and Protein Expression

Genetic potential of microbial communities can be predicted from metagenomic sequences using tools like PICRUSt [110]. However, it is better to determine gene and protein expression directly. The key is to know the whole genomic sequence of target bacterial species [6,111]. Once RNA sequencing or protein mass spectrometry is performed on total RNA or protein purified from digesta [112,113,114,115], such datasets can be repeatedly searched against the target genome and not against broad publicly available databases. Quite an elegant approach is when 16S rRNA sequencing and protein mass spectrometry are performed on the same samples. Once 16S rRNA sequencing identifies the most abundant taxa, whole genomic sequences of these taxa can be used for generation of the protein database. This can be then used for strain-specific analysis of protein mass spectra and identification of in vivo expressed proteins.

The host response to colonisation with microbiota of particular composition can be analysed as well [57,68,83,94,116,117]. Chicken gene expression at the RNA or protein level in caecal or ileal tissue can be determined in parallel with microbiota characterisation. The chicken response can be determined also in internal tissues like the liver or blood. A specific case is the detection of chicken proteins associated with gut microbiota [84,94,118]. If combined with cell sorting and DNA sequencing, bacterial species coated with particular chicken proteins, e.g., immunoglobulin or avidin, can be identified, thus allowing for a better understanding of host–microbiota interactions [84].

## 11. Digesta Metabolome and Experimental Administration of Gut Microbiota

Chickens can be analysed for low molecular weight metabolites in their digesta or in blood serum [119,120]. Not surprisingly, digesta commonly contains dipeptides, amino acids and their derivates like tyramine or γ-aminobutyric acid (GABA) [119,121,122]. Digesta also contains nucleotides, with those containing an adenine structure dominating over the others due to the presence of adenine not only in nucleic acids but also in free ATP. Multiple metabolites of plant origin, e.g., sinapine or soyasaponins, can be found in the digesta and the composition of these metabolites is highly affected by feed and microbiota composition [121,122]. However, the vast majority of low molecular weight compounds are identified only as a molecule of a particular molecular weight for which it is difficult to decide on the origin, whether it originates from feed, is released in the gut lumen by the chicken host or represents a by-product of microbial metabolism.

## 12. Conclusions

Chicks from hatcheries represent a specific model for host–microbiota studies. It is possible to characterise microbiota development in chickens from commercial settings but one should be aware that such birds represent a very specific case. Chicks from hatcheries represent a highly experimental group of chickens. Transfer of adult type of chicken microbiota to offspring opens ways towards the development of new types of probiotics. If chicks are colonised by pure cultures of individual gut anaerobes [68,69,89], new data on microbiota or host expression can be obtained [112,113,121]. Despite this, one has to be aware that transfer of complete microbiota from hens to offspring acted in evolution as a compromise between gut health, body weight increase, reproduction etc. If reproduction is not any issue in broiler fattening, transfer of complete microbiota from hens to chicks need not be directly applicable. Moreover, transfer of caecal contents may represent a biosafety risk since, besides bacteria, viruses, protozoa or fungi can be transferred as well [8,9,123]. In such cases, subcultures in nutrient broths may decrease the complexity of transferred microbiota and such microbial consortia can be better suited for administration to chickens in commercial production. Which media to use for culture [111] or whether to prepare tailored consortia from individual pure cultures of gut anaerobes are obvious topics for future studies. The simple availability of nearly unlimited numbers of chicks separated from any contact with their parents thus represents both a threat and an opportunity. The threat resides in consideration of chicks from hatcheries as controls. On the other hand, if the same chicks are inoculated experimentally with microbiota of known composition, chicks available at low cost and in essentially unlimited numbers represent a unique opportunity. There is no one biological species in which offspring are separated from their parents without the need for approval by ethics committees. When this fact is correctly understood, chickens become an exciting model for host–microbiota studies.

## Data Availability

No new data were created or analyzed in this study. Data sharing is not applicable to this article.
